# The EuRREB Core Endocrine Registry and its potential for studying transition of care in Endo-ERN centres

**DOI:** 10.1530/EC-26-0333

**Published:** 2026-10-06

**Authors:** Ana Luisa Priego Zurita, Mariya Cherenko, Jacqueline van der Blom-de Gunst, Jillian Bryce, Christopher Smythe, S Faisal Ahmed, Natasha M Appelman-Dijkstra

**Affiliations:** ^1^ Department of Medicine, Division of Endocrinology, Leiden University Medical Center, Leiden, The Netherlands, Endo-ERN (European Reference Network on Rare Endocrine Conditions) centre; ^2^ Office for Rare Conditions, Royal Hospital for Children & Queen Elizabeth University Hospital, Glasgow, UK; ^3^ Developmental Endocrinology Research Group, School of Medicine, Dentistry & Nursing, University of Glasgow, Glasgow, UK

**Keywords:** database, endocrinology, longitudinal data collection, natural history, registry, rare conditions, rare diseases, Endo-ERN

## Abstract

**Abstract:**

Natural history registries can address knowledge gaps and support the development of best practice guidelines to improve outcomes in rare conditions. The European Registries for Rare Endocrine and Bone conditions (EuRREB) Core Registry is a standardised, multi-national platform designed to collect harmonised clinical data across rare endocrine disorders and facilitate longitudinal real-world analyses. The aim of this study was to describe data collected in the Core Registry across eight endocrine main thematic groups (MTGs) during the first six years following its launch in 2019, with a particular focus on the transition period, defined as 14–25 years of age. Forty-eight centres from 20 European and non-European countries contributed data, of which 33 (69%) were members of the European Reference Network on rare endocrine conditions (Endo-ERN). A total of 3,225 unique patients were registered, with a median age of 37 years. Overall, the hypothalamic and pituitary MTG accounted for the largest number of records (*n* = 1,548). The highest proportion of patients in the transition age group was observed in the sex development and maturation MTG (66%, 445/677), followed by thyroid (33%, 86/260) and growth and genetic obesity (32%, 42/130). A total of 303 patients had an active platform account, and 85 completed patient-reported outcome measures. Clinicians completed a total of 2,390 condition-specific outcomes. These findings demonstrate the capacity of the Core Registry to generate real-world insights across rare endocrine conditions and age groups. The observed transition-age representation highlights an opportunity to strengthen structured pathways and support continuity of care across the paediatric–adult interface.

**Plain language summary:**

The European Registries for Rare Endocrine and Bone conditions (EuRREB), supported by the Endo-ERN, collects information on rare diseases to improve care. This study shares what we learned over six years, including how young people move from paediatric to adult care.

## Introduction

Rare conditions, defined in Europe as disorders affecting fewer than 1 in 2,000 individuals, compose a heterogeneous group of diseases that represent a challenge to healthcare (Rare diseases – Public Health – European Commission, last accessed: March 31, 2026). Rare endocrine conditions are heterogeneous, often systemic, and frequently present during childhood. As patients reach adolescence and adulthood, they face a particularly vulnerable phase: the transition from paediatric to adult services.

Although the transition from paediatric to adult care is widely recognised as a vulnerable phase, it remains under-studied, and the implementation of structured transition programmes across Europe remains inconsistent despite increasing guidance on best clinical practice (1, 2, 3). Recent joint clinical practice guidance from the European Society for Paediatric Endocrinology (ESPE) and the European Society of Endocrinology (ESE) emphasises the importance of structured, multidisciplinary, and patient-centred transition programmes to optimise long-term outcomes for young people with chronic endocrine conditions (4). However, a recent survey conducted by the European Reference Network on Rare Endocrine Conditions (Endo-ERN) Transition of Care Working Group demonstrated considerable heterogeneity in transition practices across European centres, with many institutions lacking formal protocols, transition coordinators, or standardised assessment tools (5). Similar findings were previously reported in a survey of Endo-ERN pituitary centres, where only 9% reported using a formal transition protocol and only one centre employed a structured transition assessment tool (3). Collectively, these findings underline the need for more consistent models of care, robust data collection, and a deeper understanding of how transition currently takes place across Europe.

Since 2017, Endo-ERN has sought to improve access to high-quality, expert care for patients with rare hormonal disorders by connecting specialist centres throughout Europe. Currently, over 105 centres from 27 countries hold membership to this network. The European Registries for Rare Endocrine and Bone conditions (EuRREB) were approved in 2018 to support the objectives of Endo-ERN by providing high-quality registries to its members and beyond. The Core Registry platform is, since 2019, the central initiative collecting standardised patient data (6). The Core Registry is a bespoke platform covering eight endocrine condition groups, the so-called main thematic groups (MTGs) that align with the categorisation followed by the Endo-ERN (Rare Endocrine Conditions – Endo-ERN, last accessed: March 31, 2026), and two additional condition groups covering bone dysplasias and systemic and rheumatologic conditions.

Natural history registries can address knowledge gaps and assist in developing best practice guidelines to improve patient outcomes in rare conditions (6, 7). Within rare endocrinology, such registries offer a unique opportunity to study variation in models of care, including the structure, timing, and quality of the transition from paediatric to adult services (8).

This study describes the data collected in the Core Registry over the six years since its launch in 2019, with a particular focus on the transition period. In light of the recently reported variation in transition practices across Endo-ERN centres (5), we also explore the potential of EuRREB as a platform to support future research on transition readiness, timing, models of care, and long-term outcomes in patients with rare endocrine conditions.

## Methods

After the launch of the Core Registry in July 2019, information about the platform was disseminated by the Endo-ERN network coordination and through scientific societies such as the European Society of Endocrinology (ESE) and the European Society for Paediatric Endocrinology (ESPE). Endo-ERN members were invited to join the platform. After the second call for ERN membership in 2021, an invitation to new members was extended. In addition to Endo-ERN member centres, centres providing care for the covered conditions but not formally designated as Endo-ERN members (non-Endo-ERN centres) were also invited to participate. To contribute to the platform, interested healthcare providers completed a self-registration form which was then reviewed and approved by the registry’s management team. Participating centres were defined as centres that had entered data into the Core Registry, irrespective of their administrative registration status within the platform.

For the categorisation of centres according to their affiliation with the Endo-ERN or other European Reference Networks (ERNs), information was retrieved from the ERN Service Directory and verified against the respective ERN websites (ERN Service Directory – Public Search, last accessed: March 31, 2026). Centres were classified as Endo-ERN, other ERN (affiliated with at least one ERN other than Endo-ERN), or non-ERN (not identified affiliation with any ERN).

### Study design and data source

This is a cross-sectional analysis of data collected through the EuRREB Core Registry, a centralised, web-based platform hosted at the Leiden University Medical Center (LUMC). The registry was established in June 2019 to enable the longitudinal collection of common and condition-specific data on patients with rare endocrine conditions across Endo-ERN and non-ERN centres. The analysis covers the first six years of activity (June 2019–June 2025).

The registry captures both clinician-reported and patient-reported outcomes and is accessible to both user roles through a secure login interface. Data entry is initiated by the clinician who creates a new patient record by completing a set of core data fields. Patients who expressed their interest in gaining access to the patient platform during the consent process receive an invitation to create an account sent by their healthcare provider via the clinician platform.

### Study population

All patients were included in MTG-level analyses, as assignment to at least one MTG is mandatory in the Core Registry. MTG-level analyses described age distributions and condition group composition, allowing patients contributing to multiple MTGs to appear more than once.

Patient-level analyses were conducted on deduplicated data. Diagnostic information is hierarchical (MTG → primary condition → specific diagnosis) and variably complete; analyses requiring greater diagnostic granularity were, therefore, restricted to records with the relevant fields completed. Diagnoses are mapped according to the Orphanet classification of rare diseases (9).

Records belonging to the Bone Dysplasia and Systemic and Rheumatological MTGs were excluded, resulting in eight MTGs analysed: adrenal; calcium and phosphate; genetic disorders of glucose and insulin homeostasis; genetic endocrine tumour syndromes; growth and genetic obesity; hypothalamic and pituitary; sex development and maturation; and thyroid.

### Variables and outcomes

The registry captures a core dataset harmonised with the set of common data elements (CDEs) (Data Dictionaries|EuRREB) defined according to the European Rare Disease Platform and FAIR principles (Set of Common Data Elements|European Platform on Rare Disease Registration, last accessed: March 31, 2026) (10) (Table 1).

**Table 1:** Variables collected in the core data and main thematic group (MTG) sections. Variables were completed for each different MTG the patient was assigned.

Variable	Answer options	Additional information
Pseudonym in the Core Registry	Generated by the system	
SPIDER	Patient’s pseudonym	CDE
Consent for Core Registry*	Opt-in; opt-out	
Date of birth*	YYYY-MM-DD	CDE
Condition group (MTG)*	Adrenal; calcium and phosphate; glucose and insulin; genetic endocrine tumours; growth and obesity; pituitary; sex development; thyroid; bone dysplasia; systemic and rheumatological	
Sex at birth*	Male; female; other; not known	CDE
Current gender	Male; female; non-binary; other; asked but unknown	
Country at birth*	All world countries	
Country of usual residence*	All world countries	
Patient follow-up status*	Patient active; patient inactive; patient care suspended; patient care terminated; deceased; unknown	CDE
Date of death	YYYY-MM-DD	CDE
Primary cause of death	Dropdown list ICD-10 codes	
Can be contacted for research purposes by clinician responsible for patient*	Yes; no; not known	CDE
Data can be shared for research purposes*	Yes; no; not known	CDE
Can be contacted for collecting patient-reported outcomes	Yes; no; not known	
Patient would like access to Core Registry*	Yes; no; not known	
e-REC ID	e-REC ID number	MTG
First contact with centre*	YYYY-MM-DD	CDE; MTG
Clinician responsible for patient*	Name of clinician responsible for patient (choose from registered users in the platform and centre)	MTG
Date of first clinical manifestations*	YYYY-MM-DD	CDE; MTG
How was diagnosis reached	Clinical; biochemistry; genetic; histology; CT; MRI; PET; USS; other; not known	MTG
Date of diagnosis*	YYYY-MM-DD	CDE; MTG
Primary condition*	Primary condition by MTG	MTG
Specific diagnosis*	Specific diagnosis by primary condition and MTG	CDE; MTG
Participation in detailed disease registry	Yes; no; not known	MTG
Detailed disease registry	Registry name	MTG
Detailed disease registry ID	Registry ID number	MTG
Biobank sample available for research*	Yes; no; not known	CDE; MTG
Biobank details	Name of biobank	CDE; MTG
Biobank patient ID	Biobank patient ID	MTG

SPIDER, secure privacy-preserving identity management in distributed environments for research; CDE, common data element; MTG, main thematic group; ID, identifier; CT, computed tomography; MRI, magnetic resonance imaging; PET, positron emission tomography; USS, ultrasound scan.

* Mandatory field.

The Core Registry currently holds seven diagnosis-specific datasets collecting longitudinal data within the endocrine field, named within the registry’s condition-specific modules (CSMs). These datasets were proposed by multidisciplinary experts, including patient advocacy group representatives. Furthermore, various patient-reported outcome measures (PROMs) on different areas are available on the platform (Fig. 1).

**Figure 1:**
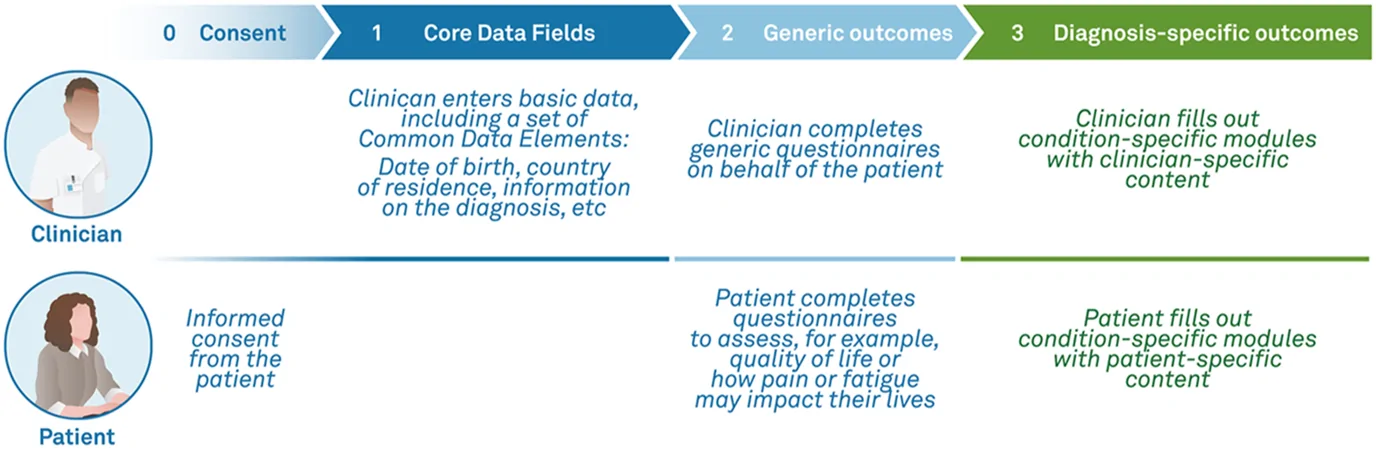
Core Registry data entry flow.

### Definition of transition age groups

For the purposes of this analysis, the transition period was defined as 14–25 years and divided into two subgroups: 14–17 years (transition planning phase) and 18–25 years (transition and early adult care phase). Patients aged < 14 years were classified as pre-transition, while those aged ≥ 26 years were considered adults beyond the transition period.

Age was summarised using the median and range between the 10th and 90th percentiles, given the non-normal distribution of age in the registry. Age distributions across predefined transition age groups were examined by main thematic group (MTG) using cross-tabulations with row percentages. Analyses were conducted at the MTG level; patients may, therefore, contribute to more than one MTG.

### Age-specific care provision

Age-specific care provision analyses were conducted at the MTG level to characterise care structures across condition groups; patients contributing to more than one MTG were, therefore, permitted to appear multiple times.

Clinician role values were categorised as paediatrician, adult physician, and other/unclear for non-missing clinician roles that could not be classified as paediatric or adult. Blanks were treated as missing and excluded from analysis.

### Statistical analysis

Analyses were performed in IBM SPSS Statistics 29. Graphs were generated using GraphPad Prism 10. Descriptive statistics were generated to summarise patient and centre characteristics, using counts, percentages, medians, and interquartile ranges as appropriate. The total number of reporting centres, patients, and diagnoses were determined overall and per MTG.

Transition-focused analyses described the distribution of transition-age patients across MTGs.

### Data management and quality control

All data are entered directly by authorised users after two-factor authentication through the EuRREB online interface. The platform includes automated mandatory fields, plausibility, and consistency checks at the time of entry. Periodic data exports are reviewed for data quality. Centres can correct or update entries at any time.

### Ethical considerations

Data were pseudonymised and handled in compliance with the General Data Protection Regulation (EU 2016/679). Informed consent for participation by all patients (or their parents/guardians for minors) was obtained by the participating centres.

## Results

### Contributing centres

Forty-eight centres from 16 countries within the European Union (EU) and four non-EU countries entered data during the defined period (Fig. 2). In 11 of the 20 countries represented, registry participation was exclusively from Endo-ERN-affiliated centres.

**Figure 2:**
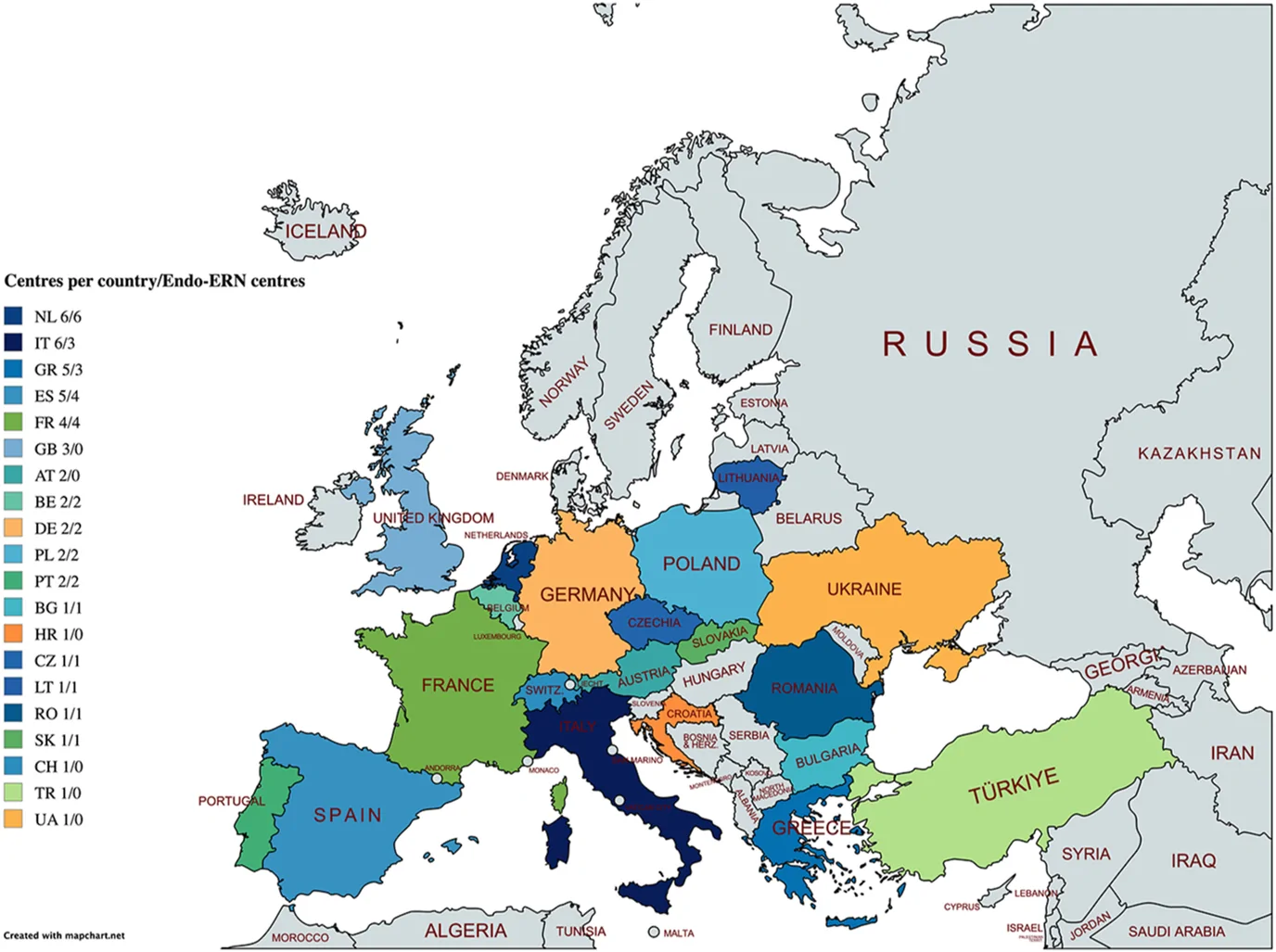
Number of centres per country and their affiliation to Endo-ERN.

Contributions were received from 48 centres. Of these, 33 (69%) were Endo-ERN members and registered 2,800/3,225 patients (87%). The median number of cases per centre and country is summarised in Table 2. Median values ranged from 1 to 205 cases per centre, with wide ranges observed in several countries, indicating marked heterogeneity in centre-level contributions.

**Table 2:** Participating centres and cases per centre by country. Centre (*n*) indicates the number of participating centres per country. The number of cases per centre is presented as median (range), based on deduplicated patient records.

Country	Centres (*n*)	Cases (median (range))
Austria	2	46 (26–65)
Belgium	2	146 (3–289)
Bulgaria	1	205
Croatia	1	2
Czech Republic	1	22
France	4	81 (6–119)
Germany	2	7 (1–13)
Greece	5	6 (1–286)
Italy	6	5 (3–655)
Lithuania	1	1
The Netherlands	6	45 (2–452)
Poland	2	57 (32–81)
Portugal	2	5 (2–8)
Romania	1	15
Slovak Republic	1	1
Spain	5	2 (1–4)
Switzerland	1	36
Türkiye	1	3
Ukraine	1	11
United Kingdom	3	1 (1–243)

Centre uptake was defined by the date of first case entry. Figure 3 illustrates the cumulative number of participating centres over time. Early uptake was primarily driven by Endo-ERN-affiliated centres, with additional increases observed from 2023. Centres affiliated with other ERNs or not affiliated with any ERN contributed from 2022, initially in small numbers but with steady growth over time. Overall, the total number of contributing centres increased consistently throughout the study period. Patient entries increased in parallel with centre participation over time.

**Figure 3:**
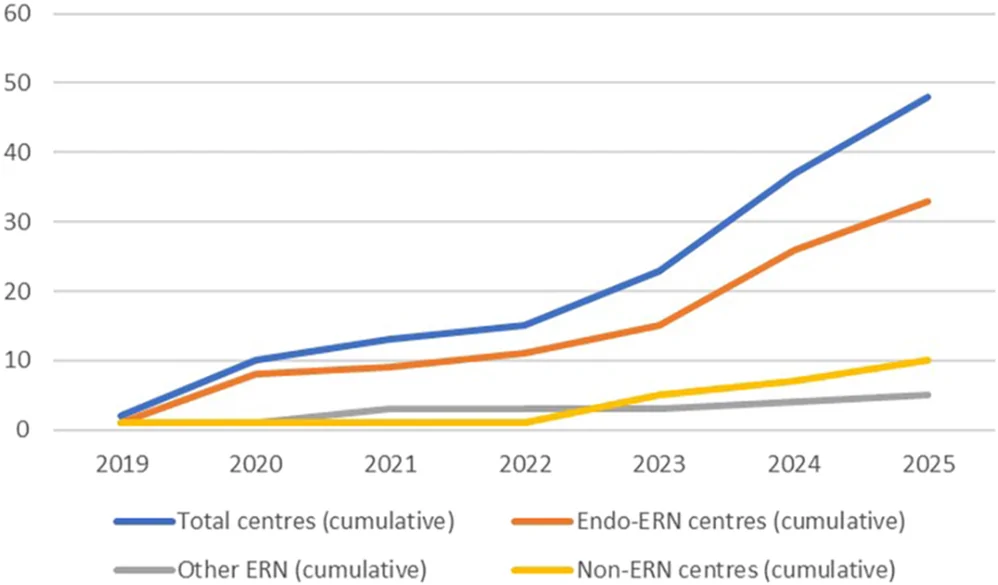
Participating centres uptake. The date of the first patient record creation was considered the start date of the participation. The category ‘Other ERN’ (European Reference Network) included members of at least one of the other 23 ERNs.

### Patient level analyses

The demographic and clinical characteristics of the registered population are summarised in Table 3. A total of 3,225 unique patients were registered. The median age at the moment of data extraction was 37 years (10th–90th percentile: 10–74). Female and male patients were equally represented in the study population, with 1,641 and 1,584 patients, respectively. The follow-up status was active for 97% of patients (2,716/2,800).

**Table 3:** Demographic and clinical characteristics of the registered population.

Characteristic	Patients
Number of unique records	3,225
Age, median (10th–90th percentile)	37 (10–74)
Sex at birth, female, *n* (%)	1,641 (51)
Endo-ERN affiliation, *n* (%)	2,800 (87)
Follow-up status, *n* (%)	2,800 (87)
Patient active	2,716 (97)
Patient inactive	56 (2)
Deceased	23 (0.8)
Patient care suspended	4 (0.1)

### MTG level analyses

Forty-five patients had more than one diagnosis across different MTGs, resulting in a total of 3,271 records. Overall, the hypothalamic and pituitary MTG accounted for the largest number of records (*n* = 1,548), followed by sex development and maturation (*n* = 677) and adrenal (*n* = 314) (Table 4).

**Table 4:** Distribution of records in the Core Registry by main thematic group and defined age groups.

Condition group	Overall, *n* (%)	Age group, *n* (%)				
		<10 years	10–14 years	15–17 years	18–24 years	≥25 years
Adrenal	314 (10)	17 (5)	10 (3)	8 (3)	33 (11)	246 (78)
Calcium and phosphate	262 (8)	43 (16)	27 (10)	16 (6)	38 (15)	138 (53)
Genetic disorders of glucose and insulin homeostasis	8 (0.2)	6 (75)	1 (13)	1 (13)	0	0
Genetic endocrine tumour syndromes	72 (2)	4 (6)	5 (7)	3 (4)	9 (13)	51 (71)
Growth and genetic obesity	130 (4)	48 (37)	36 (28)	22 (17)	20 (15)	4 (3)
Hypothalamic and pituitary	1,548 (47)	47 (3)	41 (3)	45 (3)	56 (4)	1,359 (88)
Sex development and maturation	677 (21)	96 (14)	106 (16)	150 (22)	295 (44)	30 (4)
Thyroid	260 (8)	31 (12)	34 (13)	41 (16)	45 (17)	109 (41)
Total	3,271	292 (9)	260 (8)	286 (9)	493 (15)	1,937 (59)

The proportion of adult patients was greatest within the hypothalamic and pituitary MTG with 91% (1,415/1,548), followed by adrenal with 89% (279/314) and genetic endocrine tumour syndromes. In contrast, paediatric patients predominated in genetic disorders of glucose and insulin with 100%, growth and obesity with 82% (106/130), and sex development and maturation with 52% (352/677). The highest proportion of patients in the transition age group was observed in sex development and maturation with 66% (445/677), thyroid with 33% (86/260), and growth and genetic obesity with 32% (42/130).

### Diagnosis distribution by MTG and age group

The three most frequent diagnoses by MTG and age group are summarised in Table 5. Marked age-specific differences were observed across several MTGs. For example, the hypothalamic and pituitary MTG was dominated by non-acquired pituitary hormone deficiencies in children, whereas non-functioning pituitary adenoma and prolactinoma predominated in adults. Similarly, X-linked hypophosphataemia was the leading paediatric diagnosis in the calcium and phosphate MTG, while genetic hyperparathyroidism was most common in adults.

**Table 5:** Three most frequent diagnoses by main thematic group (MTG) and age group. Where diagnoses were tied for third place, all diagnoses with the same frequency are presented. The most frequent diagnosis is presented in bold.

MTG	Children (<18 years) (*n*)	Adults (≥18 years) (*n*)
Adrenal	**21-hydroxylase deficiency (8)**; autoimmune polyendocrinopathy type 1 (2); other forms of congenital adrenal hyperplasia (2)	**Adrenocortical carcinoma (84)**; sporadic phaeochromocytoma/secreting paraganglioma (30); 21-hydroxylase deficiency (29)
Calcium and phosphate	**X-linked hypophosphataemia (47)**; iPPSD2 (10); iPPSD3 (6)	**Genetic hyperparathyroidism (72)**; X-linked hypophosphataemia (25); parathyroid carcinoma (14)
Genetic disorders of glucose and insulin homeostasis	**Beckwith–Wiedemann syndrome (2)**; congenital isolated hyperinsulinism (2); diazoxide-resistant diffuse hyperinsulinism (1); diazoxide-sensitive diffuse hyperinsulinism (1)	—
Genetic endocrine tumour syndromes	Hereditary phaeochromocytoma-paraganglioma (1); MEN1 (1); von Hippel**–**Lindau disease (1)	**Hereditary phaeochromocytoma-paraganglioma (21)**; von Hippel**–**Lindau disease (4); Carney complex (1); MEN1 (1)
Growth and genetic obesity	**Prader–Willi syndrome (30)**; Silver**–**Russell syndrome (10); Noonan syndrome (7)	**Prader–Willi syndrome (9)**; Noonan syndrome (3); melanocortin 4 receptor deficiency (2)
Hypothalamic and pituitary	**Non-acquired pituitary hormone deficiency (69)**; non-acquired combined pituitary hormone deficiency (19); acquired pituitary hormone deficiency (3)	**Non-functioning pituitary adenoma (514)**; prolactinoma (223); acquired pituitary hormone deficiency (169)
Sex development and maturation	**Transgender, female to male (85)**; transgender, male to female (44); EMS 5–8 (32)	**Transgender, female to male (164)**; transgender, male to female (37); 21-hydroxylase deficiency (9); isolated hypospadias (9)
Thyroid	**Papillary thyroid carcinoma (57)**; permanent congenital hypothyroidism (11); follicular thyroid carcinoma (6)	**Papillary thyroid carcinoma (122)**; medullary thyroid carcinoma (11); follicular thyroid carcinoma (7)

iPPSD2, pseudohypoparathyroidism type 1A; iPPSD3, pseudohypoparathyroidism type 1B; MEN1, multiple endocrine neoplasia type 1; EMS, external masculinisation score.

The number of reporting centres and registered paediatric and adult patients for each MTG is presented in Supplementary Table 1 (see section on Supplementary materials given at the end of the article).

### Age-specific care provision across MTGs and age groups

Age-specific care with predefined standards was assessed across MTGs and stratified by age group (Fig. 4). Overall, age-specific care was higher and more consistent in paediatric patients (<18 years) compared with adults. Across all MTGs, the lowest proportion of age-specific care among paediatric patients was 64%, with most MTGs demonstrating substantially higher values.

**Figure 4:**
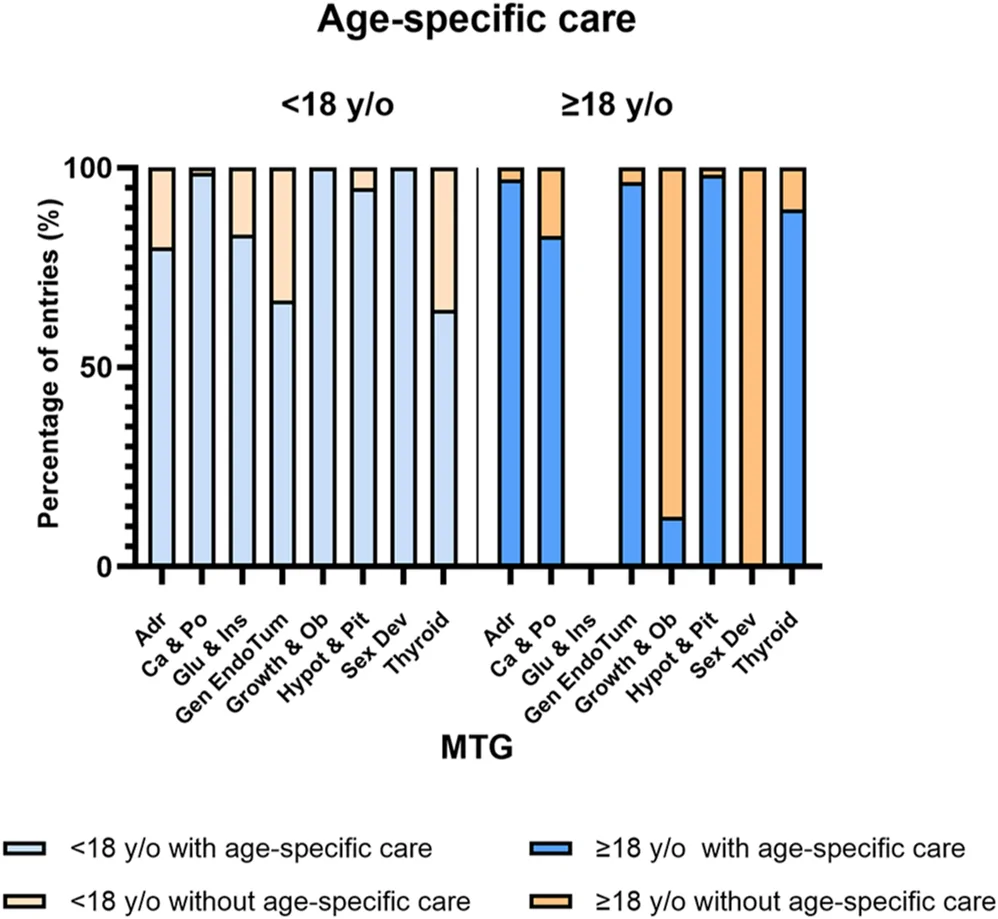
Age-specific care provision by the main thematic group. Stacked bars on the left and on the right show the proportion of paediatric and adult patients, respectively, receiving age-specific and non-age-specific care.

In contrast, concordance in adults (≥18 years) showed greater variability. While the majority of MTGs demonstrated high age-specific care rates (>80%), two MTGs exhibited markedly lower numbers (1 and 12% concordance across age groups, respectively). Notably, these MTGs are characterised by the predominance of paediatric patients within the registry.

Although age-specific care provision was high in most MTGs irrespective of ERN affiliation, Endo-ERN centres demonstrated higher homogeneous performance, whereas greater variability was observed among non-affiliated centres in selected MTGs.

### Use of the registry: patients and clinicians

Of the 3,255 registered patients, 1,183 (37%) indicated that they would like access to the Core Registry. Preference regarding access was recorded as ‘not known’ in 655 cases (20%) and was missing in 785 cases (24%). Among patients with a definitive response (yes/no), 65% expressed interest in access (Fig. 5). Of the 303 patients with an active account, 43 (14%) were children, and 260 (85.8%) were adults. Overall, 32 (11%) were of transition age, including 7 children and 25 adults.

**Figure 5:**
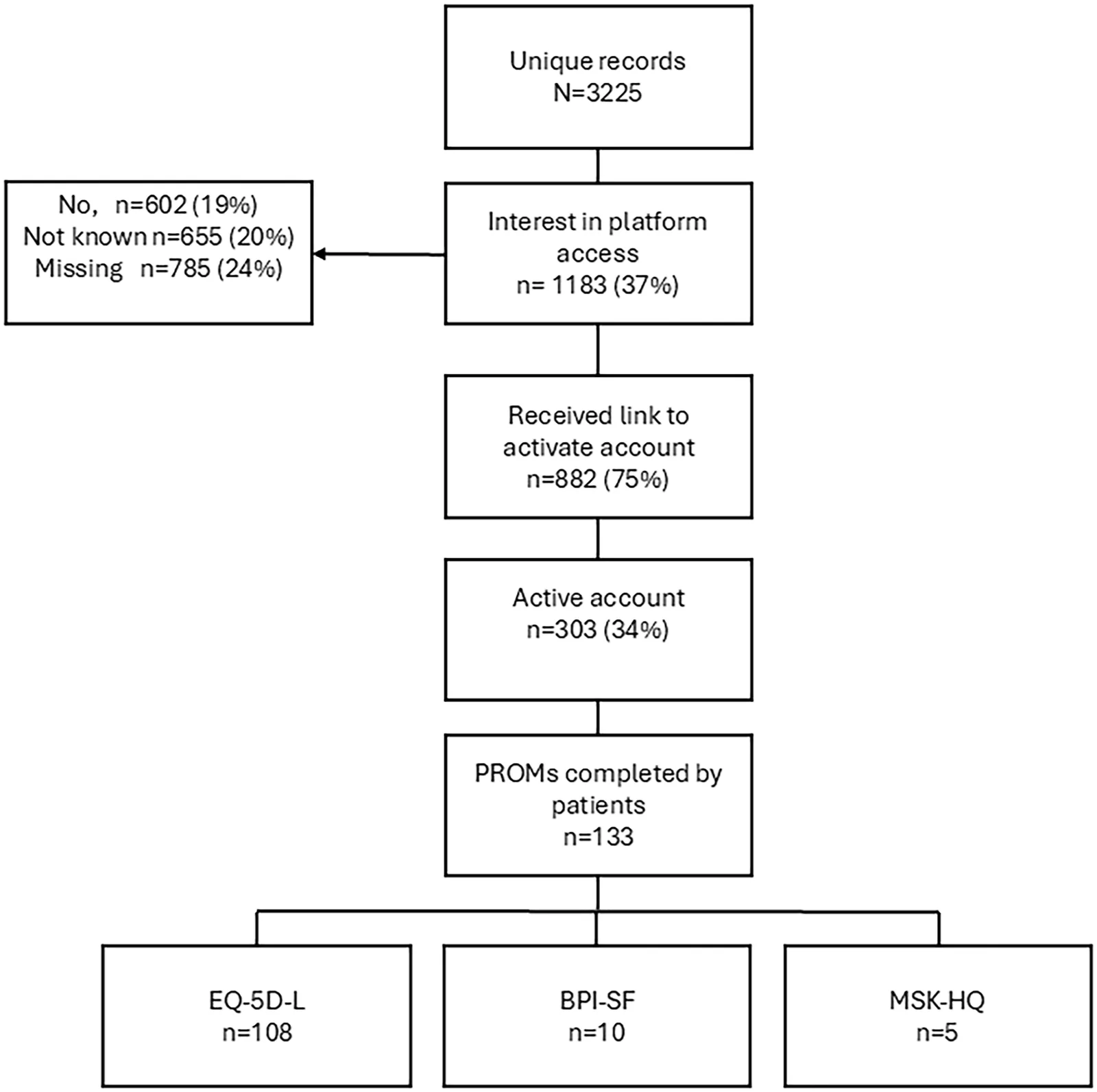
Flow of patient access and PROM completion. Flow diagram showing stages of platform access (interest, activation, and active account) and subsequent completion of PROMs. Percentages are shown relative to the preceding step; reasons for no recorded interest are indicated. EQ-5D-L: 5-level EuroQol; BPI-SF, Brief Pain Inventory – Short Form; MSK-HQ, Musculoskeletal Health Questionnaire.

A total of 177 PROMs were completed for 77 patients. Of 177 outcomes, 133 (75%) were completed by patients and 44 by clinicians. Among patients, EQ-5D-L (5-level EuroQol) was the most frequently used measure (*n* = 108), followed by BPI-SF (Brief Pain Inventory – Short Form) (*n* = 10) and MSK-HQ (Musculoskeletal Health Questionnaire) (*n* = 5) while among clinicians EQ-5D-L (5-level EuroQol) was the most used (*n* = 36), followed by the ICF (International Classification of Functioning, Disability and Health) (*n* = 7) and the BPI-SF (*n* = 2).

The number of entries across the different CSMs varied substantially. Within the calcium and phosphate MTG, the iPPSD module held 14 outcomes completed for 13 patients; the rare hypophosphataemic disorders module recorded 66 outcomes for 38 patients, while 15 outcomes for 12 patients were completed in the parathyroid carcinoma module. In the rare genetic obesity CSM, 11 outcomes were recorded for 11 patients. The pituitary tumour module showed the highest level of activity, with 1,777 outcomes completed for 612 patients. In the gender incongruence module, 326 outcomes were completed for 320 patients, and in the paediatric-differentiated thyroid carcinoma module, 181 outcomes were completed for 181 patients.

## Discussion

The Core Registry provides a scalable infrastructure that supports both epidemiological analyses and quality-of-care assessments across rare endocrine conditions. Its implementation across multiple countries demonstrates the feasibility of harmonised data collection within a heterogeneous rare disease landscape. Although EuRREB is endorsed by the European Reference Network on rare endocrine conditions (Endo-ERN), registry uptake extends beyond ERN-affiliated centres. Non-Endo-ERN centres contribute meaningfully to both centre representation and case volume, suggesting that ERNs act as early adopters and dissemination hubs, while the registry enables broader engagement beyond formal network and geopolitical boundaries. This wider uptake enhances representativeness and strengthens benchmarking potential.

Clear age-related differences in MTG predominance and diagnostic composition reflect variation in disease presentation across the life course. Supplementary Table 1 provides additional context for the observed variation in patient numbers by showing the number of centres contributing paediatric and adult patients within each MTG. Although MTGs with more reporting centres generally tended to include larger numbers of registered patients, this relationship was not uniform, indicating that patient numbers also reflect differences in disease prevalence and the scope of expertise of participating centres.

Overall, age-specific care provision was high, indicating alignment with predefined standards; however, variability across MTGs and greater heterogeneity in adult care were observed. In contrast, paediatric care demonstrated more consistent patterns. The genetic endocrine tumour syndrome MTG showed one of the lowest rates of age-specific care in the paediatric group, which may reflect the familial nature of these conditions and the likelihood that patients are managed by the same physician across age groups. Notably, in MTGs predominantly composed of paediatric patients, concordance with age-specific care was lower among adult patients, potentially reflecting differences in pathway maturity between paediatric and adult services.

In the sex development and maturation MTG, virtually all adult patients were managed by paediatric physicians; however, most were aged 18–24 years, corresponding to the transition period. A similar pattern was observed in the growth and obesity MTG, which may indicate that some patients remain under paediatric care during transition. Together, these findings highlight the complexity of defining and implementing age-appropriate care during this critical phase.

These observations align with broader challenges in rare diseases, where approximately 70% of conditions originate in childhood, and many congenital endocrine disorders present early. Improvements in survival and earlier diagnosis have increased the number of patients transitioning to adult care, placing additional demands on adult healthcare systems. The variability observed in this study underscores the need for structured transition pathways and harmonised adult care frameworks. In this context, the Core Registry functions not only as a descriptive tool but also as a benchmarking instrument capable of identifying variation, informing guideline refinement, and supporting targeted quality improvement.

The work by Shishkov *et al.* demonstrated the feasibility of using the EuRREB Core Registry to characterise transition of care in rare pituitary disease, illustrating how structured registry data can support clinically meaningful transition research (8). These findings provide an empirical basis for the development of standardised transition datasets in collaboration with EuRREB working groups.

Patient engagement represents another critical dimension. While 36% of patients expressed interest in accessing the patient platform, 20% were recorded as ‘not known’, raising questions regarding documentation practices and the systematic capture of patient preferences. Moreover, only a subset of those expressing interest had an active account, indicating a progressive loss of participants between consent and activation. Harmonisation of patient engagement processes may, therefore, strengthen participation and long-term engagement. This is particularly relevant in conditions where care pathways shift between specialities across the life course, for example from paediatric endocrinology for growth disorders to adult specialities such as osteology or rheumatology. The first condition-specific module (CSM) was launched in 2021, and in subsequent years, additional diagnosis-specific datasets have been developed by multidisciplinary groups, reflecting both investigator engagement and the strength of the network. Activity across modules and the number of patients included are substantial, underscoring the value of the Core Registry as a tool for studying the natural history of rare conditions and highlighting its potential for future research initiatives; however, variation in outcome numbers across CSMs reflects differences in module design and study group recommendations (e.g. longitudinal updates at fixed timepoints, annual reporting, or event-driven capture).

A key strength of this study is the large, multi-national dataset covering both paediatric and adult populations, enabling life-course analyses. The use of the Orphanet classification enhances diagnostic granularity and interoperability, supporting FAIR principles and facilitating data reuse. Furthermore, linking structural data (centre participation and ERN affiliation) with clinical variables (diagnoses distribution and concordance of care) enables exploration of system-level variation. As expected in a real-world, multi-centre registry, variable completeness and heterogeneity in documentation were observed. Systematic evaluation of data completeness is, however, an integral component of registry governance and quality assurance. These limitations reflect pragmatic data collection and do not diminish the value of the registry as a monitoring and benchmarking platform.

Future directions include developing transition-focused datasets, longitudinal analyses of transition, expansion of patient-reported outcomes, and structured feedback to participating centres. Integration with broader European data initiatives may further enhance interoperability and long-term sustainability. Beyond describing disease distribution, the Core Registry serves as a dynamic instrument for monitoring care patterns, benchmarking practice, identifying variation, and supporting harmonisation across European rare endocrine services. Efforts should be sustained to strengthen connections between paediatric and adult care, at both the clinician and patient levels.

## Supplementary materials



## Declaration of interest

The authors declare that there is no conflict of interest that could be perceived as prejudicing the impartiality of the research reported. SF Ahmed is the Editor-in-Chief of *Endocrine Connections*. He was not involved in the peer-review process of this paper in which he is listed as an author.

## Funding

This paper is part of the projects: 777215 (EuRRECa) and 946831 (EuRR-Bone), which have received funding from the European Union’s Health Programme (2014–2020). Since 2025, these projects have been continuing under the name ‘EuRREB’, as part of the following projects: 101156495 (Endo-ERN), 101157116 (ERN BOND), and EU4H-2023-ERN2-IBA-01. The European Commission’s support to produce this publication does not constitute endorsement of the contents which reflect the views only of the authors, and the Commission cannot be held responsible for any use which may be made of the information contained therein. As of April 1, 2021, the Consumers, Health, Agriculture and Food Executive Agency (CHAFEA) ceased to exist. The portfolio of actions managed by CHAFEA under the 3rd HP was transferred to the Health and Digital Executive Agency (HaDEA).

## Ethics statement

The project complies with the General Data Protection Regulation (EU 2016/679) and was approved by the Information Governance authorities at the NHS Greater Glasgow and Clyde Health Board and the National Research Ethics Service in the UK (Core Registry REC Reference 18//WS/0205) and later by the Medical Ethics Committee at the Leiden University Medical Center (Core Registry Protocol nWMODIV2_2024018, project ID: 130850).
