# Supplementary material for: The EuRREB Core Endocrine Registry and its potential for studying transition of care in Endo-ERN centres
Source: Endocr Connect. 2026 Oct 6;15(10):e260333. doi: 10.1530/EC-26-0333 (PMC13695395; doi:10.1530/EC-26-0333)
Supplement: Supplementary file 1 [file EC-26-0333_supplementary_table_1.pdf]

Supplementary table 1. Reporting centres per Main Thematic Group and age group

MTG	Centres total	Reporting centres <18 (n)	Patients <18 (n)	Reporting centres ≥18 (n)	Patients ≥18 (n)
Adrenal	13	8	35	11	279
Calcium & phosphate	21	13	86	18	176
Genetic disorders of glucose & insulin homeostasis	4	4	8	0	0
Genetic endocrine tumour syndromes	8	4	12	7	60
Growth & genetic obesity	10	9	106	8	24
Hypothalamic & pituitary	18	11	133	16	1415
Sex development & maturation	13	11	352	11	325
Thyroid	26	21	106	18	154

Reporting centres are defined as centres contributing at least one patient within the respective Main Thematic Group (MTG) and age category. A centre contributing both paediatric and adult patients is counted once in each age category but only once in the total number of reporting centres.
